# Microelectrode arrays in combination with *in vitro* models of spinal cord injury as tools to investigate pathological changes in network activity: facts and promises

**DOI:** 10.3389/fneng.2013.00002

**Published:** 2013-03-04

**Authors:** Miranda Mladinic, Andrea Nistri

**Affiliations:** ^1^Neuroscience Department, International School for Advanced Studies (SISSA)Trieste, Italy; ^2^Spinal Person Injury Neurorehabilitation Applied Laboratory, Istituto di Medicina Fisica e RiabilitazioneUdine, Italy; ^3^Department of Biotechnology, University of RijekaRijeka, Croatia

**Keywords:** central pattern generator, *in vitro* preparation, organotypic slices, locomotion, spinal cord network, motoneuron, excitotoxicity, microelectrode arrays

## Abstract

Microelectrode arrays (MEAs) represent an important tool to study the basic characteristics of spinal networks that control locomotion in physiological conditions. Fundamental properties of this neuronal rhythmicity like burst origin, propagation, coordination, and resilience can, thus, be investigated at multiple sites within a certain spinal topography and neighboring circuits. A novel challenge will be to apply this technology to unveil the mechanisms underlying pathological processes evoked by spinal cord injury (SCI). To achieve this goal, it is necessary to fully identify spinal networks that make up the locomotor central pattern generator (CPG) and to understand their operational rules. In this review, the use of isolated spinal cord preparations from rodents, or organotypic spinal slice cultures is discussed to study rhythmic activity. In particular, this review surveys our recently developed *in vitro* models of SCI by evoking excitotoxic (or even hypoxic/dysmetabolic) damage to spinal networks and assessing the impact on rhythmic activity and cell survival. These pathological processes which evolve via different cell death mechanisms are discussed as a paradigm to apply MEA recording for detailed mapping of the functional damage and its time-dependent evolution.

## Spinal cord injury: a major challenge awaiting new solutions

Spinal cord injury (SCI) is one of the most prominent causes of severe disability worldwide, with lifelong devastating dysfunction, and high medical and social costs. Most of the six million SCI patients worldwide are of young age and for them the chances of recovery are very low (Garbossa et al., 2012). In fact, to date there is no treatment that can restore neuronal connectivity to the injured spinal cord and re-establish function of neuronal networks responsible for standing and walking. This realization has prompted the search, in recent years, of novel technical approaches and strategies to understand the molecular changes underlying lesional processes, the functional organization of the spinal cord and its plasticity. New technologies like Microelectrode arrays (MEAs) can be useful for studying and eventually developing new strategies to treat diseases of the central nervous system (CNS) including SCI, which all represent an important burden to society and which, too often, remain incurable (WHO, 2008; Smith, 2011).

As reviewed by McDonald and Sadowsky (2002), the pathophysiology of the SCI is complex, as it begins with molecular and cellular events occurring immediately after traumatic (or non-traumatic) injury, and it continues with pathological processes that develop over hours, days, and even weeks later (so called secondary injury). Thus, after the primary injury that causes local cell damage and death, the secondary ischemia, anoxia, excitotoxicity, free-radical formation, inflammation, edema, and finally glial scar formation, all contribute to degeneration of neuronal and glial cells in the adjacent, initially-spared spinal segments (Schwab et al., 2006). Finally, aberrant plasticity with reorganization of spinal networks may occur and produce dysfunction like neuropathic pain or spasticity. The location and extension of the injury will determine the ensuing impairment of sensory and motor functions as well as persistent autonomic disabilities.

One major problem restraining current strategies to restore the lost connectivity in the spinal networks responsible for walking is our incomplete anatomical knowledge of the neuronal circuits subserving locomotion. Furthermore, our limited ability to control the fundamental mechanisms responsible for death and regeneration of the neurons in those networks is a current impediment to significant clinical progress. SCI pathological events belong to the same category of unsolved issues such is the inability of the mammalian central neurons to regenerate their fibers after injury and the impossibility for surviving neurons to replace or substitute dead postmitotic cells, with few localized exceptions (Bellenchi et al., 2013). In fact, Ramón y Cajal observed that “Once development was ended, the fonts of growth and regeneration of the axons and dendrites dried up irrevocably” (quoted by Bellenchi et al., 2013). In addition, the mechanisms involved in the pathways leading to neuronal death are incompletely understood so that specific strategies for neuroprotection are still preliminary (Kuzhandaivel et al., 2011). Very few molecules have reached clinical studies and none of them has provided effective treatment for SCI patients (Tohda and Kuboyama, 2011). The reasons for clinical failure of preclinical studies may include, besides incomplete knowledge of these processes, factors like unsuitable models, different protocols and the difficulty of detailed animal tissue analysis beyond a single time point.

Therapeutic strategies arising from animal studies have mostly focused on stem cells, which might provide trophic and immunomodulatory factors to enhance axonal growth and contrast neuroinflammation (Regenberg et al., 2009; Garbossa et al., 2012; Karimi-Abdolrezaee and Eftekharpour, 2012; Reeves and Keirstead, 2012). The possibility that they will replace dead neurons (i.e., that they will differentiate into neurons after transplantation, integrate within neuronal circuits and generate axons reaching the muscle) remains an experimental approach that needs further studies (Garbossa et al., 2012). Various degrees of functional recovery has been lately obtained in SCI models following transplantation of stem cells (Abematsu et al., 2010; Cizkova et al., 2011; Nori et al., 2011; Nakajima et al., 2012). Recent clinical studies (Kumar et al., 2009; Pal et al., 2009; Sharma et al., 2012) have shown the safety of these procedures in man, although functional benefit to patients is not systematically proven and may depend on lesion severity and stage (Tohda and Kuboyama, 2011; Garbossa et al., 2012). Even when substantial axonal sprouting across the spinal lesion is achieved, there is no significant functional recovery (Lu et al., 2012), implying that damage may derive from processes extending beyond the mere repair of damaged fibres. Schira et al. (2012) show, in the adult rat, moderate locomotor improvement after spinal cord hemisection concomitant with local injection of human umbilical stem cells and an immunosuppressant drug. Perhaps future research should first focus on the mechanisms of intact CNS to understand and maximize the potential of stem cell treatment (Illis, 2012).

These results should be aimed at preserving surviving neurons and optimizing their function by exploiting neuroplasticity (Oudega and Perez, 2012). Such goals demand precise knowledge of the network topography, connectivity, molecular structure, metabolism, and physiology. Thus, in the recent years the development of *in vitro* preparations of the spinal cord that readily generate electrically oscillatory cycles with the hallmarks of locomotor patterns, offers unrivalled opportunities for advance in this field. The MEA technology can provide longlasting recording of the basic network properties essential for motor rhythms.

## A blueprint for the locomotor CPG

Locomotion is the result of the complex coordinated activity of many groups of muscles, commanded by motoneurons in the ventral horn of the spinal cord, where they represent the output elements of an extended network. The circuits responsible for locomotion are driven by intrinsic spinal networks, collectively called central pattern generator (CPG; Grillner et al., 1998; Heckmann et al., 2005; Kiehn, 2006; Boulenguez and Vinay, 2009; Rossignol and Frigon, 2011) because these circuits can organize rhythmic locomotor activity in the absence of supraspinal and sensory inputs. Several studies have also shown that motoneurons are not merely passive actuators of rhythmicity as they play a significant role in modulating locomotor patterns via an interplay of their own membrane conductances in the neonatal (MacLean et al., 1997) and adult (Manuel et al., 2012) spinal cord.

Recent studies of mouse genetics have provided substantial advances in the classification of propriospinal neurons involved in locomotion by identifying distinct neuronal subtypes that contribute to certain phases and properties of the locomotor pattern (Kiehn, 2006; Brownstone and Wilson, 2008; Goulding, 2009; Grillner and Jessell, 2009; Ziskind-Conhaim et al., 2010). Hence, the overall organization of the locomotor CPG, though still incompletely understood, is seen less and less as a black-box structure because key cellular members and the pattern of synaptic interconnections between them begin to be elucidated in detail and validated with computer modeling (Zhong et al., 2012). An interesting model supported by a body of experimental evidence indicates that the motor control organization consists of two fundamental structures with hierarchical arrangement, namely a rhythm generating top module (responsible for periodic clock-like discharges) and a pattern generating bottom structure (creating alternation between flexor and extensor and between left and right limb muscle commands; McCrea and Rybak, 2008). It is also noteworthy that, despite their different phylogenetic classification, all mammals, including man, use similar processes of locomotion (Grillner, 2011) made by a series of elementary programs termed “locomotor primitives” (Dominici et al., 2011). This finding clearly shows that the use of locomotor networks of non-human mammals can provide important data of wide relevance to human studies.

While rhythm generation is considered to be dependent on glutamatergic excitatory neurons, pattern generation controlling left-right alternation and flexor-extensor alternation is thought to require the participation of inhibitory GABAergic/glycinergic interneurons (Talpalar et al., 2011). In mammals, the hindlimb locomotor networks are distributed throughout the lumbar enlargement: L1–L5 in rodents, L3–L5 in cats (Kiehn, 2006). In humans, evidence for the existence of the CPG that controls locomotion comes from studies of patients with complete spinal lesion, in whom involuntary stepping movements with reciprocal activity of limb muscles can be elicited by spinal cord focal stimulation, most effectively at L2–L3 level (Rosenfeld et al., 1995; Duysens and Van de Crommert, 1998; Dietz, 2003; Dimitrijevic, 2012). The deficits in the spinal networks controlling locomotion and in their afferent inputs are involved not only in the pathophysiology of the SCI, but also in other neurological disorders such as Parkinson's disease or stroke sequelae (Dietz, 2003; Meacham et al., 2011).

## *In vitro* preparations to study spinal locomotor networks

Isolated spinal cord preparations, deprived of afferent or descending inputs, can be maintained *in vitro* to generate electrical oscillatory cycles which possess all the hallmarks of locomotor patterns (Kiehn, 2006). Nonetheless, the absence of limbs makes necessary to refer to this pattern as fictive locomotion, which is the most convincing evidence for the existence of the spinal CPG capable of generating rhythmic, walk-like outputs (Duysens and Van de Crommert, 1998). Like real locomotion, this pattern comprises left/right signal alternation at homosegmental level, and homolateral alternation between flexor and extensor motor pools, all occurring with regular periodicity. *In vitro* this phenomenon is usually recorded from ventral roots of the lumbar region. When synaptic inhibition is pharmacologically blocked, a clock-like synchronous pattern spontaneously emerges from motor pools (Bracci et al., 1996): this activity is termed disinhibited bursting, and represents a useful tool to investigate the properties of basic rhythmicity. It is interesting that disinhibited bursting can be generated by a topographically limited network that comprises a ventral horn quadrant only (Bracci et al., 1996), while fictive locomotion needs at least three intact lumbar segments (Kjaerulff and Kiehn, 1996), demonstrating the requirement for an extended network to express all the functional characteristics of locomotion. Thus, it should be noted that slice or culture preparations are intrinsically unable to generate fictive locomotion, but they do produce basic rhythmic patterns likely arising from the same, albeit anatomically reduced, locomotor networks. Indeed, the strong interactions between fictive locomotion and disinhibited bursting (Beato and Nistri, 1999) suggest that they are both generated by the same networks or at least by networks with extensively overlapping elements.

Isolated spinal cord preparations of neonatal rodents (isolated from the brainstem to the cauda equina) show good survival *in vitro* and, have, therefore, been widely used to study fictive locomotion (Kiehn, 2006; Mladinic et al., 2013). Chemical substances can be applied to produce fictive locomotion that persists for a long time without showing fatigue (Cazalets et al., 1992; Beato and Nistri, 1998; Kiehn et al., 2000; Marchetti et al., 2001; Kiehn, 2006; Nistri et al., 2006; Taccola and Nistri, 2006; Juvin et al., 2007; Cowley et al., 2010). In particular, as first shown by Kudo and Yamada (1987), fictive locomotion can be induced by application of N-Methyl-D-aspartate (NMDA) and/or 5-hydroxytryptamine (5-HT; serotonin) (Kudo and Yamada, 1987; Cazalets et al., 1992; Beato and Nistri, 1998; Pearlstein et al., 2005), or other excitatory transmitters (Cowley and Schmidt, 1994), or by increasing the extracellular K^+^ concentration (Bracci et al., 1998). To improve *in vitro* survival and experimental access to networks, spinal cord organotypic cultures, that maintain the basic cytoarchitecture of the *in vivo* tissue and synaptic connectivity, have been developed. Even though these cultures cannot generate locomotor-like patterns, they exhibit spontaneous rhythmic activity which propagates to the whole preparation, conserving basic components of rhythm generation (Streit et al., 2001).

## MEA recording reveals important mechanisms for CPG activity

The CPG functional structure implies a series of interconnected oscillators with regular discharges organized into a rhythm (Selverston and Moulins, 1985). This concept applies also to the locomotor CPG. Hence, to analyze simultaneously the activity of topographically-distinct neurons in a spinal network, the extracellular multisite recording technology performed with slice cultures of embryonic rat spinal cords grown on multielectrode arrays, is a particularly useful approach to study the process of generation and propagation of rhythmic activity (Streit, 1993; Streit et al., 2001; Tscherter et al., 2001; Avossa et al., 2012). These authors used MEAs containing 68 electrodes arranged on a hexagonal grid (inter-electrode distance = 200 nm). Channels showing activity as fast voltage transients (corresponding to action potentials of neuronal cell bodies or axons) supplied the electrophysiological data that were analyzed with dedicated software (Streit et al., 2001). In such slice cultures, most spontaneous activity emerges from discrete “hubs” where there is a high probability of detecting neuronal firing that can spread with depolarizing waves to engulf the whole preparation and even induce contraction of co-cultured skeletal muscle fibres (Tscherter et al., 2001; Czarnecki et al., 2008). Certain spinal neurons, including motoneurons, show intrinsic spiking activity that can be recorded in the absence of synaptic inputs, a phenomenon that makes such cells as candidates to initiate rhythmicity when a few of them fire synchronously (Darbon et al., 2002; Czarnecki et al., 2008, 2009). Co-culturing spinal slices with skeletal muscle has shown that the intrinsic firing of motoneurons may contribute to the activation of population bursts through cholinergic positive feedback loops (Magloire and Streit, 2009). Furthermore, by combining MEA recording with single cell patch clamping, it has been possible to identify cellular mechanisms responsible for burst generation like the persistent sodium current (Darbon et al., 2004), and the modulatory role of serotonin in rhythmicity (Czarnecki et al., 2009).

These studies suggested a common rhythm-generating network that can be activated in organotypic slices by different protocols that comprise disinhibition (with pharmacological blockers; Ballerini and Galante, 1998; Ballerini et al., 1999) or network excitation evoked with high K^+^ and low Mg^2+^ (Streit et al., 2001). Moreover, the patterns of rhythmic activity similar to those in spinal slice cultures are recorded in dissociated cultures of rat embryonic spinal cord and grown on MEA, showing that the patterns of rhythmic activity seen in spinal slice cultures can be reproduced in randomly assembled networks: this result suggests that rhythmic activity is controlled by the interplay of intrinsic neuronal activity and recurrent excitation in neuronal networks without the need for specific architecture (Streit et al., 2001).

## MEA in SCI studies: present and future

The CPG networks that are genetically programmed to express locomotion often remain relatively intact following SCI which simply causes their disconnection from descending command centers (Meacham et al., 2011). These networks can be activated by electrical stimulation applied to the surface of the spinal cord (Courtine et al., 2009), or even more efficiently with fine electrodes implanted in the spinal tissue (Mushahwar et al., 2007; Bamford and Mushahwar, 2011). These data suggest that it would be feasible to use MEA to investigate the properties of spinal networks after SCI and how chemical or electrical stimulation can facilitate their oscillatory discharges. It is noteworthy that, using spinal organotypic cultures from mice expressing the genetic phenotype G93A of hereditary lateral amyotrophic sclerosis, a selective dysregulation of synaptic transmission was demonstrated (Avossa et al., 2006). Likewise, MEAs were used to study the electrophysiological activity of motoneurons in spinal cord slices from mouse models of spinal muscular atrophy (Zhang et al., 2010). Furthermore, a battery of biomarkers may be employed to correlate functional damage with cell type loss in organotypic cultures (Avossa et al., 2006; Cifra et al., 2012). Since conventional arrays of rigid microelectrodes can be substituted with elastomer-substrate MEA technology, this stretchable MEAs (five electrode configuration) can be wrapped around the isolated spinal cord to stimulate spinal tracts (without any penetrating injury) in the attempt to activate fictive locomotion (Meacham et al., 2011).

In our laboratory, isolated spinal cord preparations of neonatal rats as well as spinal organotypic cultures have been used to investigate the molecular mechanisms involved in the delayed cell death of locomotor networks after acute experimental injury (Kuzhandaivel et al., 2011). Novel experimental paradigms have been developed to mimic the consequences of strong or weak SCI lesions, taking as end point the functional activity of locomotor networks in relation to the number and topology of surviving cells (Taccola et al., 2008). The use of organotypic cultures is predicted to allow an extended follow up of functional and structural damage evolution well beyond the *in vitro* survival of slices or isolated preparations. This is feasible study since, at least within the first 24 h after the primary lesion, the damage evolution progresses with similar properties in isolated preparations and organotypic cultures (Mazzone et al., 2010). Our model has already been shown to be suitable for preclinical testing of neuroprotective drugs selectively directed toward dysregulated mechanisms leading to neuronal or glial cell death (Margaryan et al., 2010; Kuzhandaivel et al., 2010a,b, 2011; Nasrabady et al., 2011a,b; Sámano et al., 2012). In addition, nanotechnologies (that employ carbon nanotubes; Bareket-Keren and Hanein, 2012; Parpura et al., 2013) in association with MEA recording may be tested for the ability to promote network repair and the functional outcome after experimental SCI. Electrophysiological data have already indicated an unexpected increase in synaptic efficiency when spinal networks are interfaced with these new materials (Cellot et al., 2011; Fabbro et al., 2012).

**Figure 1:**
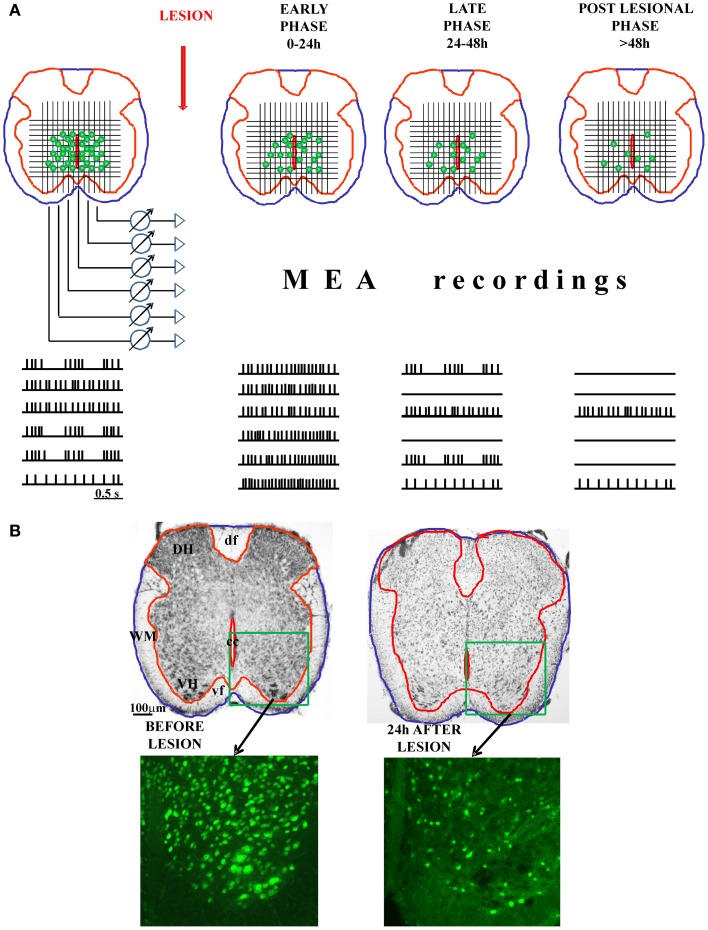
**Possible application of MEA on spinal cord slices to study the functional progression of the injury and the involvement of individual neurons in the control of locomotion.** In the panel **(A)**, we show the schematic presentation of freshly-cut spinal cord slices, before and after experimental injury (with three different phases of the injury: early, late, and post-lesional). The functional progression of the injury could be monitored (in the real time) using MEA technology. Namely, the MEA containing tens of electrodes could be arranged on a grid to cover the central-ventral region of the spinal cord, which contains the neurons involved in the control of locomotion. MEA would allow the recording of the electrical activity (action potentials) of numerous neuronal cells in the CPG zone. With the progression of the injury and the death of the cells, the number of spiking neurons would decline, giving the possibility to correlate the remaining functional activity with the histological analysis panel **(B)**. The combination of MEA recordings and the topographical mapping of the live, electrically active neurons in the lesioned spinal cord slices could define precisely the spinal network oscillators. DH, dorsal horn; VH, ventral horn; WM, white matter; df, dorsal funiculus; vf, ventral funiculus; cc, central canal.

Alternatively, the MEA could also be applied to freshly-cut spinal cord slices, in analogy to studies of rat neocortical and hippocampal brain slices (Becker et al., 2012) set up to examine the mechanisms underlying anesthetic (isoflurane)-induced excitation. One might envisage monitoring, in real time, the onset and distribution of experimental SCI by recording the progressive loss of neuronal activity and assess its time dependent evolution as proposed in Figure 1A. This approach would be useful if implemented in conjunction with tests for delayed recovery of rhythmicity either spontaneously or after applying neuroprotection (or neurorepair) protocols. Ultimately, this approach might transfer to a MEA recording chamber the experimental paradigm used with the isolated spinal cord preparation for which detailed immunohistochemical data are already available (Figure 1B). An obvious advantage of this approach would be the possibility to create a functional map to guide future experiments with the aim of identifying potential mechanisms of cell resilience to damage through more precisely defined circuits of spinal network oscillators.

### Conflict of interest statement

The authors declare that the research was conducted in the absence of any commercial or financial relationships that could be construed as a potential conflict of interest.
